# Teaching well-being at scale: An intervention study

**DOI:** 10.1371/journal.pone.0249193

**Published:** 2021-04-14

**Authors:** David B. Yaden, Jennifer Claydon, Meghan Bathgate, Belinda Platt, Laurie R. Santos

**Affiliations:** 1 Department of Psychiatry and Behavioral Sciences, Johns Hopkins University School of Medicine, Baltimore, Maryland, United States of America; 2 Poorvu Center for Teaching and Learning, Yale University, New Haven, Connecticut, United States of America; 3 Yale School of Medicine, Biological and Biomedical Sciences, New Haven, Connecticut, United States of America; 4 Department of Psychology, Yale University, New Haven, Connecticut, United States of America; Simon Fraser University, UNITED STATES

## Abstract

Courses that teach evidence-based interventions to enhance well-being are a public health tool that could be used to improve mental health in the population. We compared the well-being of six cohorts of adult students before and after they completed one of two massive open online courses: *The Science of Well-Being* (*N* = 581; 441; 1,228) and a control course, *Introduction to Psychology* (*N* = 677; 480; 1,480). Baseline well-being levels were equivalent across all six samples. Students in both courses increased in their well-being from baseline to follow-up in all three samples (*p* < .001); however, at follow-up, students in *The Science of Well-Being* course had higher subjective well-being than the control course (sample 1: *r* = .18, *d* = .37, *p* < .001; sample 2: *r* = .21, *d* = .43, *p* < .001; sample 3: *r* = .19, *d* = .38, *p* < .001). Overall, across three samples, we found that students who completed either of these online psychology courses increased in their well-being––but that students in *The Science of Well-Being* course showed greater improvement. These findings suggest that large free online courses that teach evidence-based approaches to well-being could positively impact mental health at large scales.

## Introduction

The US population is facing historically high levels of mental illness [1–3], which has left healthcare providers and public health advocates looking for effective solutions and preventative measures [4]. While effective treatments exist for many mental illnesses, many of these remain underutilized due to issues related to access, finances, equity, and stigmas related to obtaining services. As such, interventions with a broader reach may be needed to address mental health issues at the population level.

Further, many have argued that the definition of positive mental health should go beyond the reduction of mental illness and incorporate the promotion of well-being. According to the World Health Organization: “health is a state of complete physical, mental and social well-being and not merely the absence of disease or infirmity” [5, 6]. That is, mental health is not only about reducing mental illness, but also involves proactively enhancing subjective well-being (SWB). SWB is defined as a person’s appraisal of their own internal mental states, usually including emotions and an overall assessment of one’s life as a whole [7]. Improving subjective well-being throughout the population would provide resilience against mental illness and be an end unto itself. However, this raises the question: can well-being be enhanced at scale?

A growing empirical literature suggests that engaging in relatively brief Positive Psychology Interventions (PPIs), defined as activities intended to enhance well-being, can indeed effectively improve SWB [8–10]. These PPIs include activities like counting ones’ blessings, expressing gratitude to others, using one’s signature strengths, and increasing social connection [8]. While meta-analyses demonstrate mixed results, PPIs do appear to exert a reliable, albeit small, enhancing effect on well-being, with updated aggregations of effect sizes ranging from *r* = .1 to *r* = .17 [11–13].

A number of colleges and universities have developed academic courses designed to teach students about the science of well-being and the possible positive outcomes of engaging in evidence-based PPIs. These courses typically involve teaching students about the rationale, methods, and findings of scientific research related to well-being. Crucially, such courses also allow students to take part in experiential exercises based on evidence-based PPIs and other activities known to improve well-being (e.g., exercise, sleep, and mindfulness meditation). Some universities have made these course offerings available online as part of massive open online courses (MOOCs) that are open to the public.

With some university MOOCs attracting millions of learners at a time, there is potential for academic courses on the topic of well-being and their associated PPIs to affect SWB and improve mental health at a very large scale. Unfortunately, little work to date has explored whether PPIs are able to positively impact SWB, or whether such interventions work in an online format. In the current study, we direct test whether a MOOC on the science of well-being can positively improve SWB. We chose to test this question in one popular MOOC on the science of happiness—*The Science of Well-Being* course taught by Yale University on the Coursera.org platform. *The Science of Well-Being* (hereafter *SoWB*) has attracted over 3.3 million learners since its inception in 2018, making it one of this platform’s most popular courses to date. We evaluate changes in SWB associated with this *SoWB* course compared to a control course from Yale University, *Introduction to Psychology*. Both courses were administered on the same MOOC platform over the same periods of time. We analyzed three different time spans of data for both courses, hypothesizing (see pre-registration for one of three samples: https://osf.io/27ngc) that well-being would increase in the *SoWB* course more than in the control course.

## Methods

### Study design

We measured SWB in learners of two different courses on Coursera, a popular massive open online course (MOOC) platform. Participants in Yale’s *The Science of Well-Being* (*SoWB*) and Yale’s *Introduction to Psychology* (control) MOOC courses were asked to consent to participate in a study measuring changes in their subjective well-being (SWB). We assessed students’ SWB prior to the start of each course and after the conclusion of each course. Courses are administered on an on-going basis, as the lectures are all recorded and assignments are automated, so that students can proceed at their own pace. Data for both courses were downloaded across identical time-spans. We downloaded our first sample from August 26^th^, 2018 to May 15^th^, 2019. We then preregistered our predictions for a second cohort (on the Open Science Framework: https://osf.io/27ngc) and downloaded a second sample of data for both courses (May 16^th^, 2019 to November 5^th^, 2019). We then downloaded a final cohort (May 11^th^, 2020 to August 7^th^, 2020) in order to correct some methodological issues with the first two studies and to examine whether positive effects would also obtain during the time of the Covid-19 pandemic. This study was approved by the Yale University Institutional Review Board. All experiments were performed in accordance with relevant guidelines and regulations. When providing their informed consent to participate in the research study, all participants agreed that they were 18 years or older.

### Study participants

We applied several exclusion criteria to participants who consented to participate in the study. First, we required that participants complete our well-being assessment only once before and once after the class. Participants were provided with a Qualtrics link before and after the course; however, some participants took the assessment multiple times, perhaps in order to explore how the measure works or to share it with friends or family. Future studies should make it clearer that the survey should only be taken once for research purposes and then provide another version of the survey to try taking multiple times. These participants were excluded, as this would break the assumption of independence. Second, participants must have completed the entire SWB survey to have their data included in our sample. We only included one brief scale as an outcome measure, so missing data in this instance would be indicative of failing to devote a minimal degree of attention to the measure. Third, participants who reported completing the entire 10-week course in under 2 weeks were excluded, as it would not be possible to complete the assignments properly due to their stipulated length. After applying these exclusion criteria, we obtained a sample of *N* = 581 for the *SoWB* course and *N* = 677 for the control course in the first time-span, *N* = 441 for the *SoWB* course and *N* = 480 for the control course in the second time-span, as well as *N* = 1,228 for the *SoWB* course and *N* = 1,480 for the control course in the third time-span.

### Outcome variables

We examined SWB using the PERMA Profiler, a well-validated multi-dimensional psychometric self-report measure [14]. This measure provides an overall well-being variable composed of several sub-components, including: positive emotions, engagement, relationships, meaning, and accomplishment [15]. The current study only used the main 15 PERMA items. This measure also includes reverse-scored ‘filler’ items intended to break response bias, which were not scored in this study. Items were presented on a 0–10 point Likert scale. The overall well-being variable was the outcome of interest in this study. The Cronbach’s alpha for this scale was adequate at all time points across both courses (*SoWB*: time-span 1 baseline α = .87, follow-up α = .86; time-span 2 baseline α = .93, follow-up α = .94; time-span 3 baseline, α = .93, follow-up α = .95. Control: time-span 1 baseline α = .85, follow-up α = .86; time-span 2 baseline, α = .92, follow-up α = .94; time-span 3 baseline α = .93, follow-up α = .93).

### Independent variables

Study condition was the independent variable. The two study conditions consisted of the experimental group, those enrolled in *The Science of Well-Being* (*SoWB; experimental*) course and the control group, those enrolled in *Introduction to Psychology* (*IP; control*). Participants were not randomized into condition, but baseline assessments were compared across courses to ensure that initial levels of well-being did not differ across groups.

### Statistical analysis

Analyses were performed using R. *P* < .05 was considered statistically significant. Differences between courses at both baseline and at follow-up were assessed using independent sample, un-paired t-tests. Differences within participants from baseline to follow-up in both courses were assessed using paired t-tests. Effect sizes were derived using means and standard deviations. These analyses were conducted on each of the three samples.

### Participants

In the first sample (time-span 1), a total of 1,876 participants consented in the *SoWB* course and 8,289 participants consented in the *control* course. We excluded participants who took the pre assessment multiple times (349 in *SoWB*, 689 in control), those with missing data in the assessment measure (85 in *SoWB*, 610 in control), and participants who finished the 10-week course in under 2 weeks (30 in *SoWB*, 83 in control). We also removed individuals (831 in *SoWB*, 6230 in control) for the following (a) those who took the pre, but not post survey (b) those who took the post, but not pre survey (c) those who took the pre survey once but the post survey more than one time. After applying exclusion criteria and merging the pre and post datasets, the sample resulted in *N* = 581 for the *SoWB* course and *N* = 677 for the control course. When comparing those who consented but were dropped by these various criteria, there were no differences across overall well-being at baseline between the *SoWB* course, X^2^ (111, *N* = 813) = 96.301, *p* = 0.838, and the control, X^2^ (140, *N* = 7533) = 135.87, *p* = 0.58.

In the second sample (time-span 2), after pre-registration, the same exclusion criteria were applied. A total of 1,527 participants consented in the *SoWB* course and 9,225 participants consented in the *control* course. We again excluded participants who took the pre assessment multiple times (275 in *SoWB*, 748 in control), those with missing data in the assessment measure (55 in *SoWB*, 1258 in control), learners who finished the 10-week course in under 2 weeks (33 in *SoWB*, 94 in control). We also removed individuals (723 in *SoWB*, 6645 in control) for the following (a) those who took the pre, but not post survey (b) those who took the post, but not pre survey (c) those who took the pre survey once but the post survey more than one time. After applying exclusion criteria and merging the pre and post datasets, the sample resulted in *N* = 441 for the *SoWB* course and *N* = 480 for the control. When comparing those who consented but were dropped by these various criteria, there were no differences in overall subjective well-being at baseline for those who completed the *SoWB* class, X^2^ (111, *N* = 554) = 122.656, *p* = 0.212. We did find a marginally significant difference in overall subjective well-being in the control group at baseline between those who completed the course and those who dropped the course X^2^ (146, *N* = 7721) = 175.984, *p* = 0.046.

In the third sample (time-span 3), the same exclusion criteria were applied. A total of 5,639 participants consented in the *SoWB* course and 6,081 participants consented in the control course. We excluded participants who took the assessment multiple times (2,664 in *SoWB*, 310 in control), those with missing data in the assessment measure (390 in *SoWB*, 126 in control), learners who finished the 10-week course in under 2 weeks (423 in *SoWB*, 437 in control). For this time-span, because we were able to obtain demographic information, we excluded those who did not provide demographics. We also removed individuals (934 in *SoWB*, 3728 in control) for the following (a) those who took the pre, but not post survey (b) those who took the post, but not pre survey (c) those who took the pre survey once but the post survey more than one time. After applying exclusion criteria and merging the pre and post datasets, the sample resulted in *N* = 1,228 for the *SoWB* course and *N* = 1,480 for the control. When comparing the well-being at baseline of those who consented but were dropped by these various criteria and those who completed the course, there were no differences across overall well-being between the SoWB course, X^2^ (124, *N* = 1903) = 136.95, *p* = 0.201, and the control, X^2^ (149, *N* = 24763) = 175.04, *p* = 0. 071.

Barriers in the course platform prevented us from obtaining demographic information in the first two samples, but we were able to record this information in the third sample. There were substantially more females than males in both courses. For *SoWB*, the gender breakdown was: Female = 75% (924), Male = 24% (294), Non-binary .01% (10). For the control course, the gender breakdown was: Female = 74% (1091), Male = 25% (377), Non-binary .01% (12). There was no significant difference in gender enrollments across the two courses, X^2^ (1, *N* = 2686) = 0.846, *p* = 0.358. The *SoWB* course consisted of students who were older than the control course (*SoWB*, M = 37.43, SD = 13.52; Control, M = 26.72; SD = 11.21, *p* < .001). In terms of ethnicity, the *SoWB* course was: White 61% (749), Asian 18% (227), Hispanic 12% (152), Other 5% (67), Black 3% (31), and Native American .2% (2). The ethnicity of the control course was: Asian 45% (665), White 29% (435), Hispanic 13% (195), Other 9% (128), Black 4% (54), Native American .2% (3).

We also gathered Covid-19 related information from participants in the third sample, the only time-span that overlapped with the Covid-19 pandemic. We asked participants whether they have tested positive for Covid-19 on a scale from 0 (definitely not) to 4 (definitely yes). There was more self-reported incidence of Covid-19 in the *SoWB* course (*SoWB*, M = .62, SD = .97, Control, M = .42, SD = .81, *p* < .001). We asked, in two separate questions, whether Covid-19 was impacting participants’ 1) personal life and 2) professional life on a scale of 0 (none) to 4 (a great deal). The impact of Covid-19 on personal life was higher for the *SoWB* course students than control (*SoWB*, M = 2.32, SD = 1.02; Control, 1.99, SD = 1.05, *p* < .001). The impact of Covid-19 on professional life was higher for the *SoWB* course students (*SoWB*, M = 2.53, SD = 1.17; Control, M = 2.30, SD = 1.19, *p* < .001). Lastly, we asked whether participants were taking the course due to Covid-19 (same response options as previous questions). More students in the *SoWB* were taking the course due to Covid-19 than control (*SoWB*, M = 1.93, SD = 1.38; Control, M = 1.34, SD = 1.31, *p* < .001).

## Results

**Fig 1:**
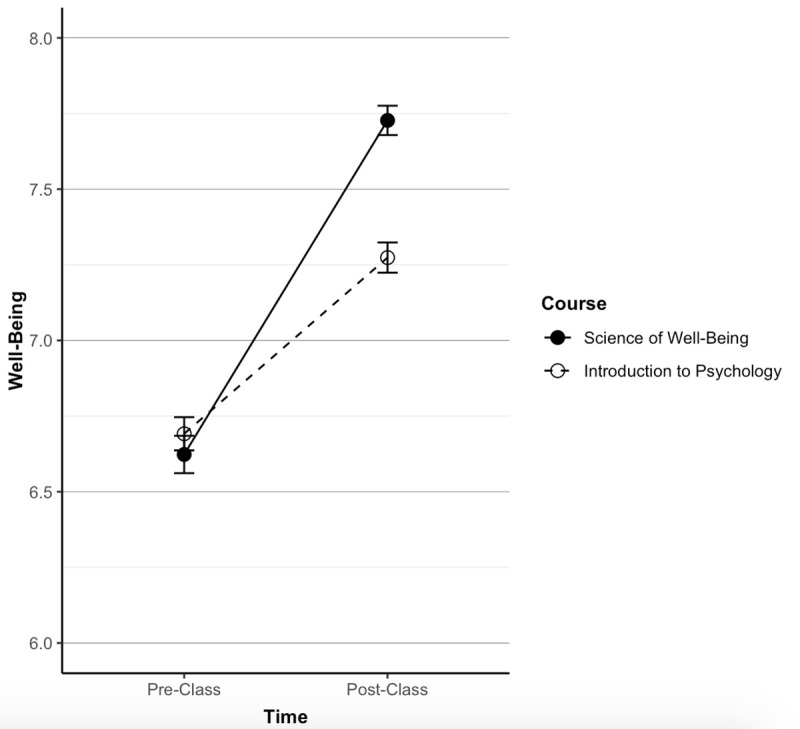
Comparison of well-being across courses in sample one. In the first time-span, the *SoWB* course (*N* = 581) and the control course (*N* = 677) did not differ at baseline but were different at follow-up (*SoWB*, M = 7.73, SD = 1.17; Control, M = 7.27, SD = 1.30, *r* = .18, *d* = .37, *p* < .001).

In the first sample (time-span 1), subjective well-being did not differ between the courses at baseline (*SoWB*, M = 6.62, SD = 1.49; Control, M = 6.69, SD = 1.43, *p* = .408). At follow-up, participants differed in terms of well-being across the two courses, with the *SoWB* course students showing significantly higher well-being scores than Control (*SoWB*, M = 7.73, SD = 1.17; Control, M = 7.27, SD = 1.30, *r* = .18, *d* = .37, *p* < .001). Participants in both courses improved from baseline to follow-up (paired t-tests from baseline to follow-up within both *SoWB* and Control were both *p* < .001). Overall, participants in both courses improved, but participants in the *SoWB* course improved more than Control. These results supported our hypothesis (see Fig 1).

**Fig 2:**
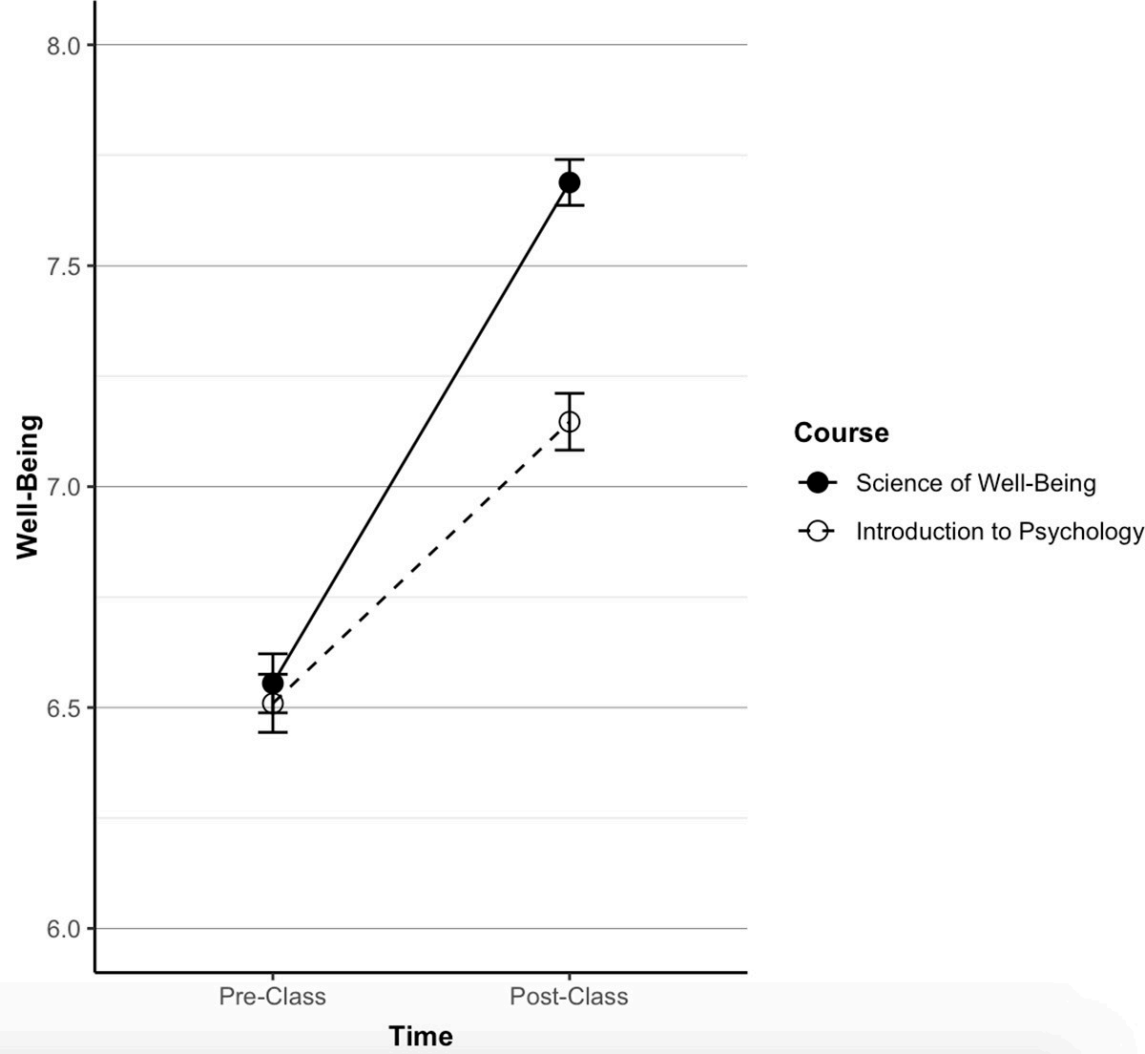
Comparison of well-being across courses in sample two. In the second time-span, the *SoWB* course (*N* = 441) and the control course (*N* = 480) did not differ at baseline but were different at follow-up (*SoWB*, M = 7.69, SD = 1.09; Control, M = 7.15, SD = 1.41, *r* = .21, *d* = .43, *p* < .001).

We replicated these results in the second sample (time-span 2) after pre-registering our hypothesis on OSF (https://osf.io/27ngc). Well-being did not differ between the courses at baseline (*SoWB*, M = 6.55, SD = 1.40; Control, M = 6.51, SD = 1.44, *p* = .628). At follow-up, participants in the *SoWB* course had higher well-being (*SoWB*, M = 7.69, SD = 1.09; Control, M = 7.15, SD = 1.41, *r* = .21, d = .43 *p* < .001). As in the first sample, students within both courses increased in well-being from baseline to follow-up (paired t-tests between baseline and follow-up in both courses were *p* < .001), but those in the *SoWB* course increased more than the control (see Fig 2).

**Fig 3:**
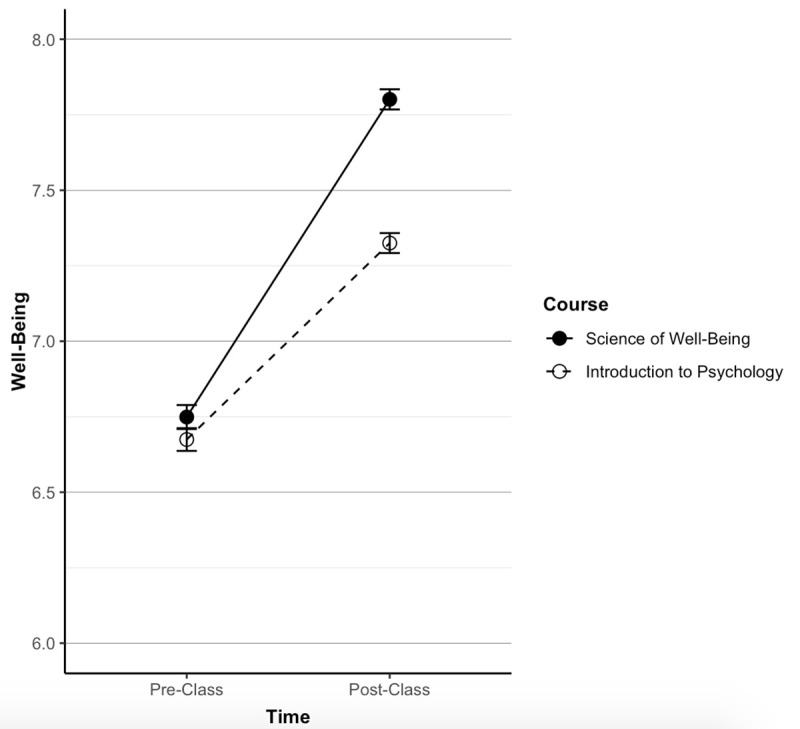
Comparison of well-being across courses in sample three. In the third time-span, the *SoWB* course (*N* = 1,228) and the control course (*N* = 1,480) did not differ at baseline but were different at follow-up (*SoWB*: M = 7.80, SD = 1.17, Control: M = 7.33, SD = 1.27, *r* = .19, *d* = .38, *p* < .001).

The results remained the same in the third, larger sample which was run during the COVID-19 pandemic. Well-being did not differ at baseline (*SoWB*, M = 6.75, SD = 1.39; Control, M = 6.67, SD = 1.45, *p* = .174). Participants within both courses increased in well-being from baseline to follow-up (*p* < .001), but when comparing between groups participants in the *SoWB* course had higher well-being than control (*SoWB*, M = 7.80, SD = 1.17; Control, M = 7.33, SD = 1.27, *r* = .19, *d* = .38, *p* < .001) (see Fig 3).

## Discussion

We found that participants who took *The Science of Well-Being* course increased in their subjective well-being more than a control *Introduction to Psychology* course. To our knowledge, this is the first study to examine the effects of a massive open online course on subjective well-being. Our findings add to the body of evidence that subjective well-being can be reliably enhanced through a combination of psychoeducation and positive interventions, and also provides evidence that such courses can take place online for free at very large scales.

These findings fit into a growing consensus that positive psychology interventions can exert relatively small but reliable increases in subjective well-being [11–13]. Importantly, however, our findings extend this work demonstrating that interventions that have previously been done in academic contexts—such as for adolescents [16] or with medical students [17]—can also work in the context of a MOOC. In doing so, our results provide new evidence that PPIs that take place in the context of free online classes can cause improvements in well-being. The results of our sample in the third time-span suggests that such positive effects still occur even during the challenging period of the COVID-19 crisis.

Taken together, these results suggest that well-being can be taught and that this can be done for free at large scales. When the various benefits of increased subjective well-being are considered, such as reduced risk of mental and physical health problems, this finding has the potential to make an impact on public health initiatives. These findings may particularly apply to academic contexts and may provide a tool to help ameliorate the rise of mental health issues in campus contexts. However, MOOCs allow any adult to participate in college-level courses (not just current college students), which leads us to believe that the implications for this new public health tool may be far-reaching. Indeed, with millions of learners enrolling in freely available online classes, there is an opportunity to make small improves at very large scales through the use of these course-based interventions. In the context of pandemics, such courses may provide a useful public tool to ameliorate the negative mental health impact due to quarantines that may increase social isolation.

## Limitations

This study had a number of limitations. First, participants were not randomly assigned across conditions. While we found that participants in both courses had the same levels of subjective well-being at baseline, it is possible that students in the *SoWB* course were more motivated to improve their well-being over the period of the course. Unfortunately, we were unable to blind the content taught in these courses and thus this explanation cannot be ruled out. Future studies could attempt to randomly assign participants to a well-being course versus a control course, yet even this design would be unable to blind learners to the content that they are learning.

A second limitation concerns the fact that a large number of students did not complete the course, did not complete the well-being assessments, and/or did not consent to participate in the study. This high rate of attrition, while problematic for the inferences that we can draw from this study, is not unusual in the context of online MOOC courses [18]. However, future studies might attempt to seek out samples in which a higher rate of completion occurs across all participants. Lastly, demographic information was not available for the first two samples. Demographic information is optional in Coursera and most students choose to remain anonymous. We addressed this issue by implementing additional, study-related demographic measures in the third time-span.

A third limitation concerns the question of why participants showed significant levels of improvement in the *SoWB* course. The *SoWB* class differed in several ways from the control introductory psychology class. First and perhaps most importantly, *SoWB* learners were asked to select one (from 8 possible PPI interventions taught during the course) and then complete their selected weekly PPI interventions as part of the class. Past work has shown that such interventions allow for improvements in SWB [8–10], and thus it is possible that these interventions alone account for the differential well-being improvements we observed in our studies. But our *SoWB* course also differed from the control course in terms of the specific academic content covered in lectures; while the control class provided a broad survey of psychology findings, the *SoWB* class covered findings specifically relevant to the science of well-being. With the present results, it’s difficult to know which aspects of these differences between the courses played the causal role–the interventions or the course content. Indeed, future studies might vary the content across studies more carefully to better assess whether the PPIs, academic content, or both is/are having a positive impact on learner’s well-being.

Finally, our study gathered follow-up data well-being measures shortly after the completion of each course. This raises the possibility that the well-being effects we observed are short-lived. Future studies could continue to track student well-being in order to determine the longevity of these well-being effects. Such studies would also allow researchers to track participants in order to determine if these effects persist.

## Conclusion

The present study demonstrated that well-being can be enhanced by taking a large-scale, free, online course. We found that participants reported enhanced well-being after taking the *SoWB* course relative to a control psychology class and that such effects emerged despite the fact that the class was administered online and at large scales. These results suggest that individuals who are exposed to academic content on the science of well-being and who engage in evidence-based practices (PPIs) can indeed increase their subjective well-being. Importantly, our findings demonstrate that freely available online courses could potentially impact mental health at large scales, and thus could become an important tool for public health initiatives aimed at improving population-wide mental health outcomes.
